# Supplementary material for: SMARCA4 loss is synthetic lethal with CDK4/6 inhibition in non-small cell lung cancer
Source: Nat Commun. 2019 Feb 4;10:557. doi: 10.1038/s41467-019-08380-1 (PMC6362083; doi:10.1038/s41467-019-08380-1)
Supplement: Supplementary file 1 — Supplementary Information [file 41467_2019_8380_MOESM1_ESM.pdf]

Supplementary Information

SMARCA4 loss is synthetic lethal with CDK4/6 inhibition in non-small cell lung cancer

Xue et al

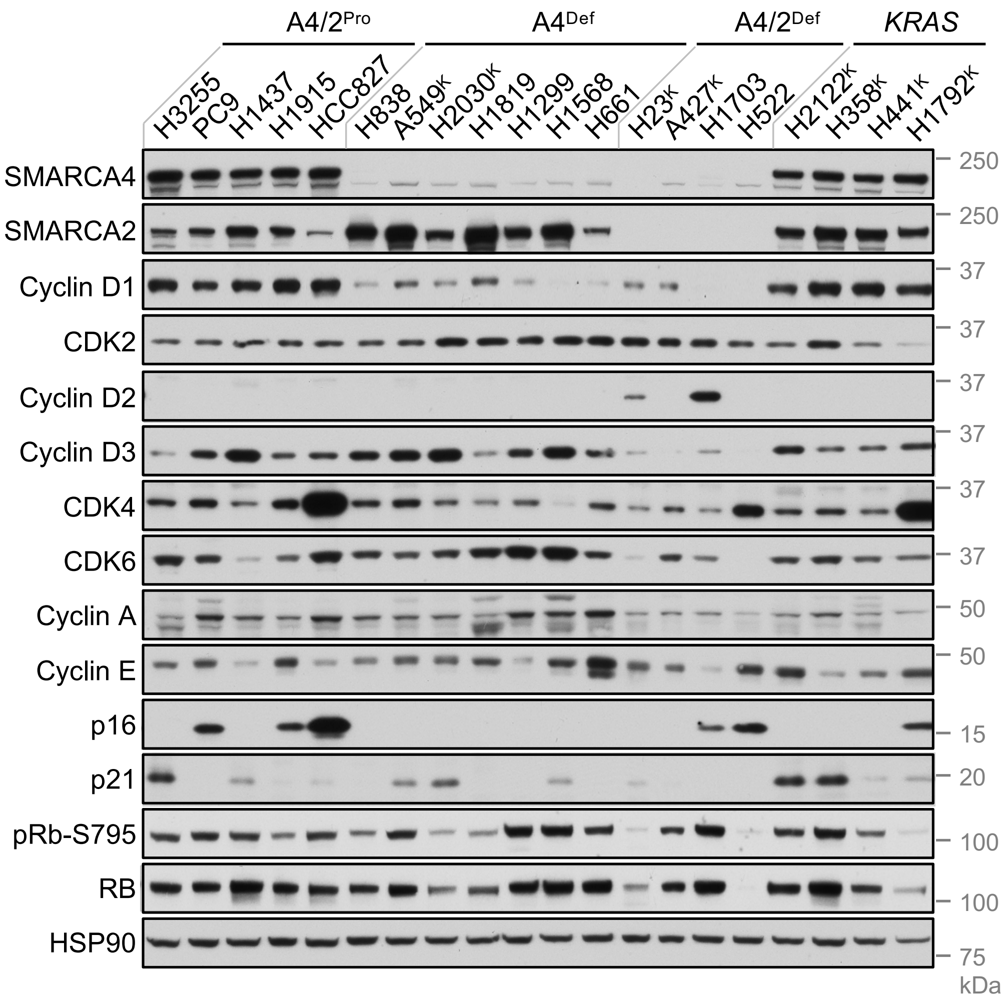

Supplementary Figure 1 | Expression of relevant cell cycle genes in a panel of NSCLC cell lines

Western blot analysis of SMARCA4, SMARCA2 and key cell cycle regulators in a cell line panel with different SMARCA2/4 status as indicated. A4: SMARCA4; A4/2: SMARCA4/2; Pro: proficient; Def: deficient; K: *KRAS* mutation. HSP90 was used as la loading control.

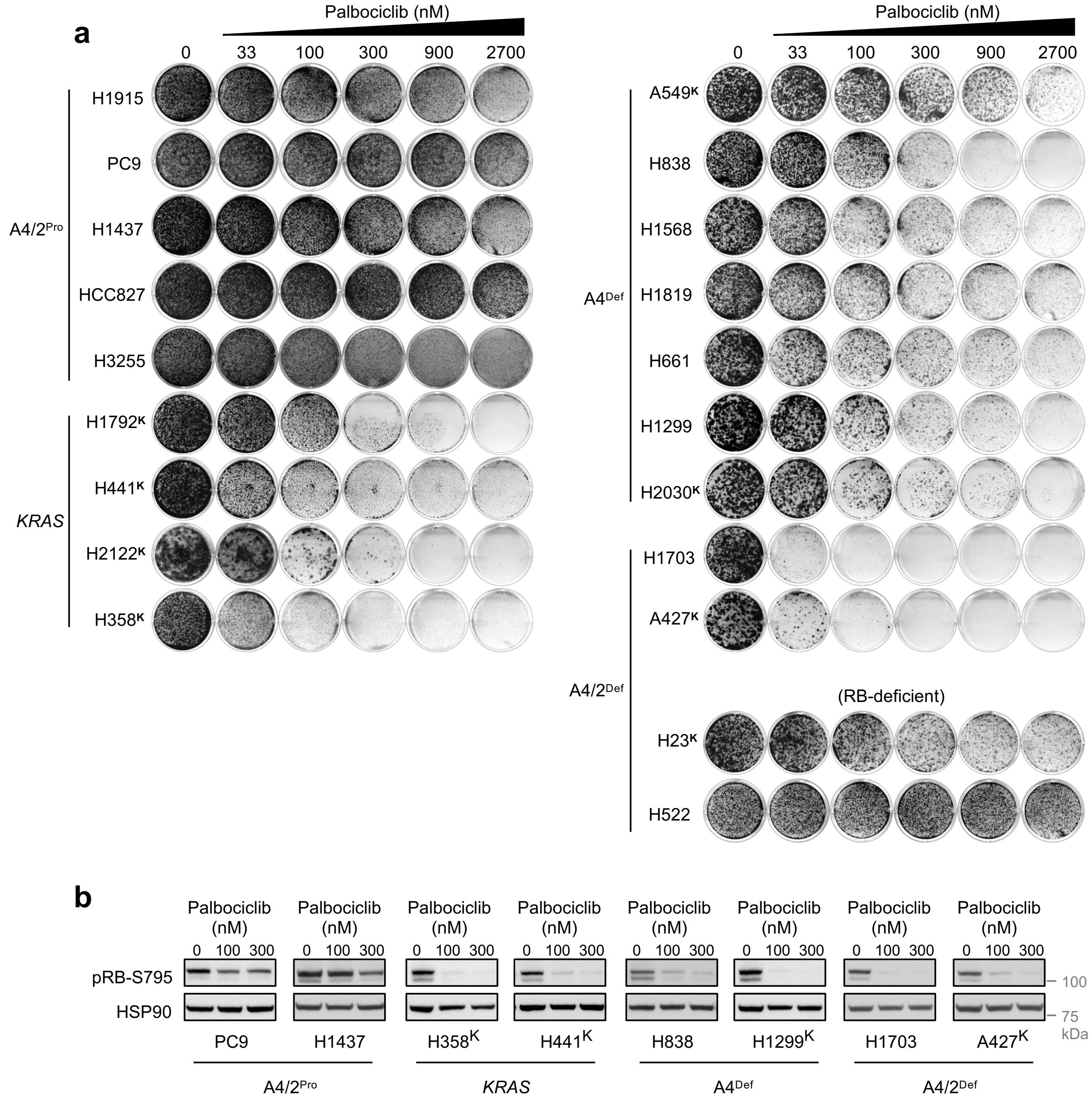

Supplementary Figure 2 | SMARCA4-deficient NSCLC cells are highly sensitive to the CDK4/6 inhibitor palbociclib

(**a**) Colony-formation assays in a NSCLC cell line panel with different SMARCA2/4 status as indicated. Cells were cultured in the absence or presence of palbociclib at the indicated concentrations for 10-14 days. For each cell line, all dishes were fixed at the same time.

(**b**) Palbociclib treatment suppresses RB phosphorylation in SMARCA4 deficient or KRAS mutant cells but not SMARCA4/2 proficient cells. Levels of pRB-S795 in cells treated with palbociclib for 24 h were documented by western blot analysis.

A4: SMARCA4; A4/2: SMARCA4/2; Pro: proficient; Def: deficient; K: *KRAS* mutation.

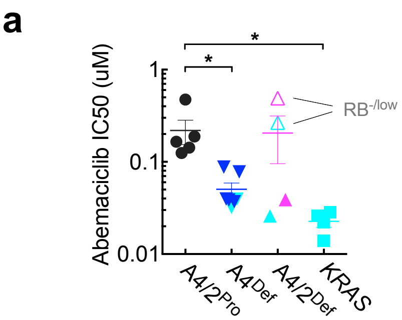

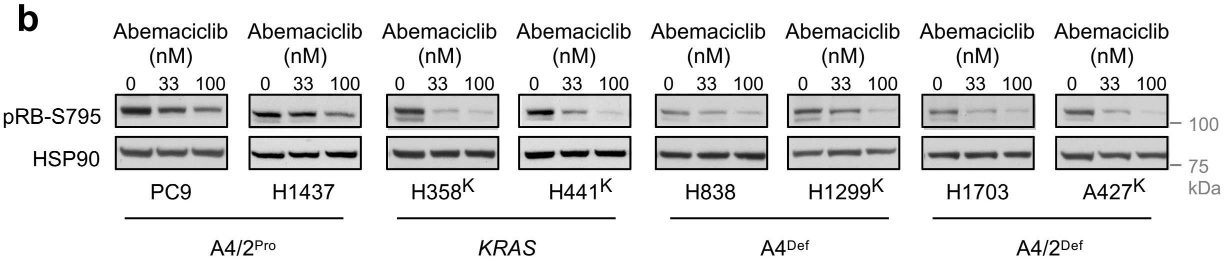

Supplementary Figure 3 | SMARCA4-deficient NSCLC cells are highly sensitive to the CDK4/6 inhibitor Abemaciclib

(**a**) Half maximal inhibitory concentration (IC_{50}) of abemaciclib in the cell line panel described in Figure 1 and Supplementary Figure 2 was evaluated by cell viability assays using CellTiter Blue. Empty triangles indicate cell lines with RB deficiency. Turquoise color indicates cell lines with *KRAS* mutation. Error bars: mean ± s.d. of biological replicates (n=3). * $p < 0.05$.

(**b**) Abemaciclib treatment suppresses RB phosphorylation in SMARCA4 deficient or KRAS mutant cells but not SMARCA4/2 proficient cells. Levels of pRB-S795 in cells treated with palbociclib for 24 h were documented by western blot analysis.

A4: SMARCA4; A4/2: SMARCA4/2; Pro: proficient; Def: deficient; K: *KRAS* mutation.

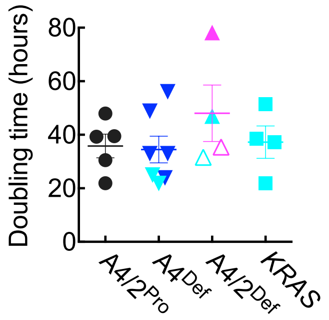

Supplementary Figure 4 | CDK4/6 inhibitors sensitivity of NSCLC is not correlated with their proliferation rate

Doubling time of the NSCLC cell lines in the panel presented in previous figures as determined from growth assays using phase-contrast live-cell imaging with an Incucyte Zoom System. Empty triangles indicate cell lines with RB deficiency. Turquoise color indicates cell lines with *KRAS* mutation. Error bars: mean ± s.d. of biological replicates (n=3).

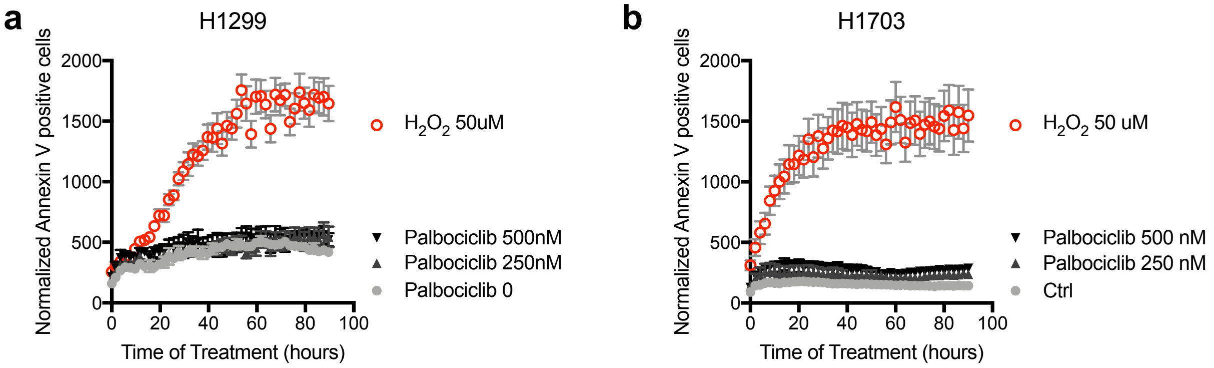

Supplementary Figure 5 | Palbociclib treatment in SMARCA4-deficient NSCLC cells does not induce cell death by apoptosis

Time course of Annexin V staining with palbociclib treatment in H1299 (**a**) and H1703 (**b**). Cells were seeded in 96-well plates with medium containing IncuCyte Annexin V Red Reagent for apoptosis and imaged every 4 hours for a total of 96 hours using the Incucyte Zoom System. The number of Annexin V-positive cells was normalized to cell confluency per well measured by phase contrast imaging. Hydrogen peroxide (H_2O_2) was used as a positive control for apoptosis induction. Error bars represent mean ± standard error of mean (s.e.m.).

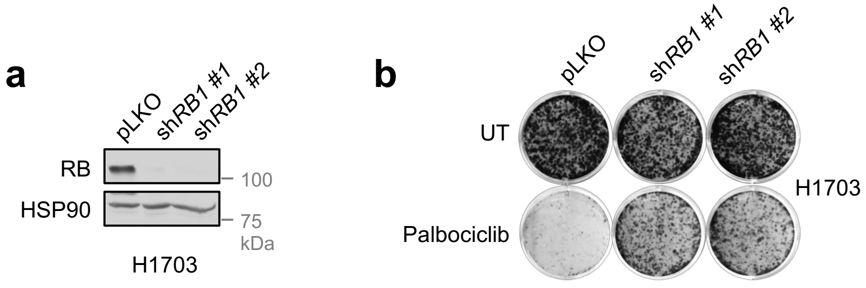

Supplementary Figure 6 | RB knockdown in SMARCA4/2-dual deficient H1703 cells confer strong drug resistance to palbociclib

(a), Western blot analysis of RB expression level in H1703 cells expressing pLKO control or two independent shRNAs targeting RB. HSP90 was used as a loading control. (b), Colony-formation assay of the cell lines described in **a)** cultured in the presence or absence of 100 nM palbociclib.

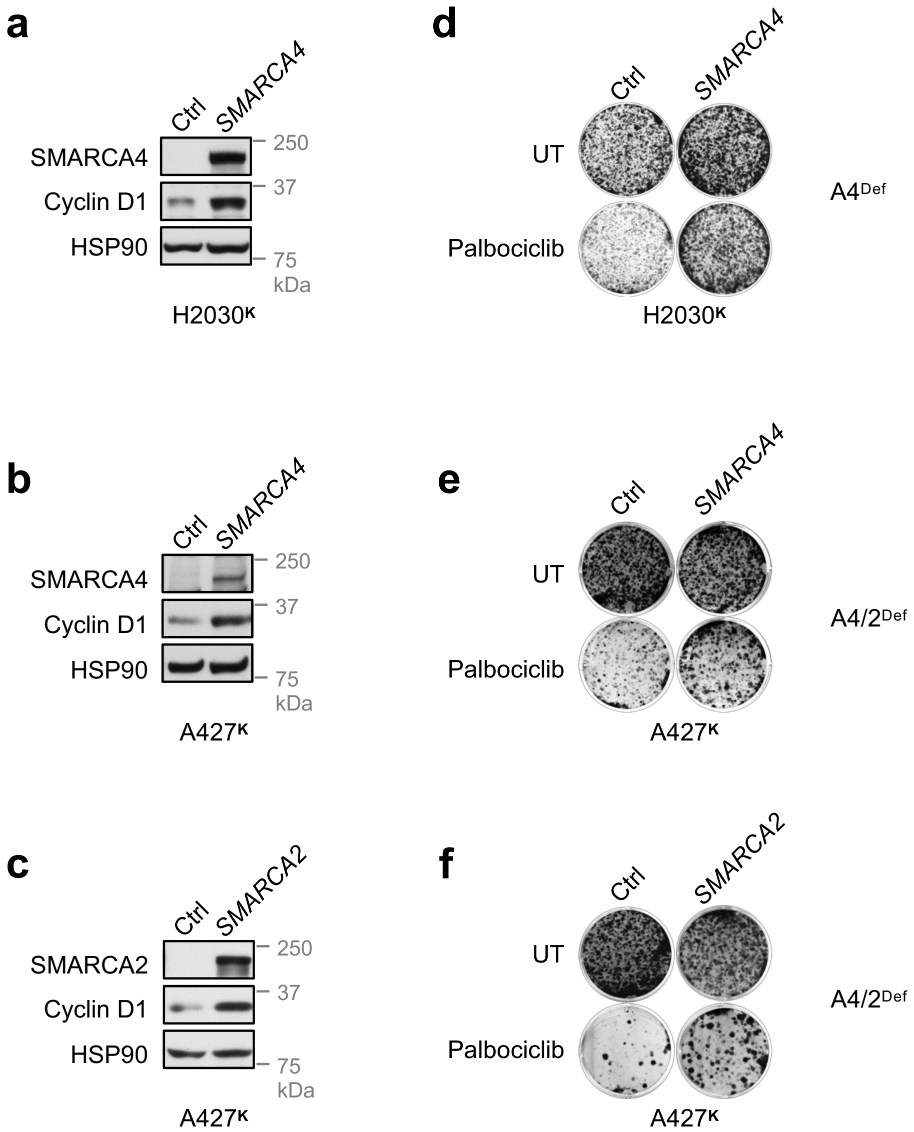

Supplementary Figure 7 | Restoration of SMARCA4 and SMARCA2 confers drug resistance to palbociclib in SMARCA4-deficient NSCLC cell lines with *KRAS* mutations

SMARCA4 restoration in *KRAS* mutant, SMARCA4-deficient cell line H2030 (**a, d**) upregulates cyclin D1 expression (**a**) and confers drug resistance to palbociclib (**d**). SMARCA4 (**b, e**) or SMARCA2 (**c, f**) restoration in *KRAS* mutant, SMARCA4/2-dual deficient cell line also upregulates cyclin D1 expression (**b, c**) and confers drug resistance to palbociclib (**e, f**).

(**a-c**) Western blot analysis for SMARCA4 or SMARCA2, cyclin D1 and HSP90 in the cells described above. (**d-f**) Colony-formation assay of the cells described above cultured in the presence or absence of palbociclib (H2030, 300 nM; A427, 100 nM).

A4: SMARCA4; A4/2: SMARCA4/2; Pro: proficient; Def: deficient; K: *KRAS* mutation.

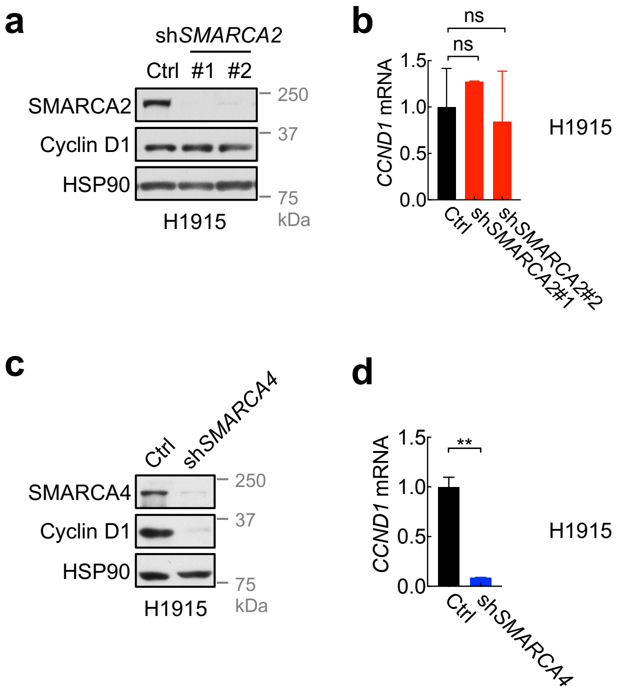

Supplementary Figure 8 | Knockdown of *SMARCA4* but not *SMARCA2* suppresses cyclin D1 expression in SMARCA4/2-proficient NSCLC cells

H1915 cells were infected with pLKO control or shRNAs targeting SMARCA4 (**a, b**) or SMARCA2 (**c, d**) and cyclin D1 expression was measured by Western blot (**a, c**) or RT-qPCR (**b, d**).
a, b *SMARCA2* knockdown in H1915 cells does not suppress cyclin D1 protein (**a**) or mRNA (**b**) expression.
c, d, *SMARCA4* knockdown in H1915 cells suppresses cyclin D1 protein (**c**) and mRNA (**d**) expression. Error bars represent mean ± standard error of mean (s.e.m.); two-tailed *t*-test; ns, not significant; ** $p < 0.01$.

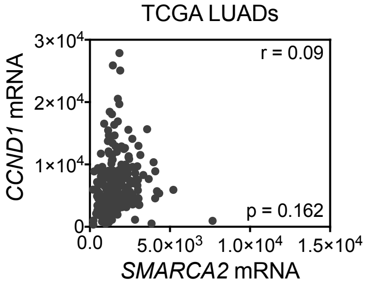

Supplementary Figure 9 | Correlation of *CCND1* and *SMARCA2* mRNA expression in lung adenocarcinomas (LUADs).

Correlation of *CCND1* and *SMARCA2* mRNA expression in LUADs of The Cancer Genome Atlas (TCGA; n = 230) cohort, without selecting for *SMARCA4* mutations. r, Pearson correlation coefficient; p, p-value.

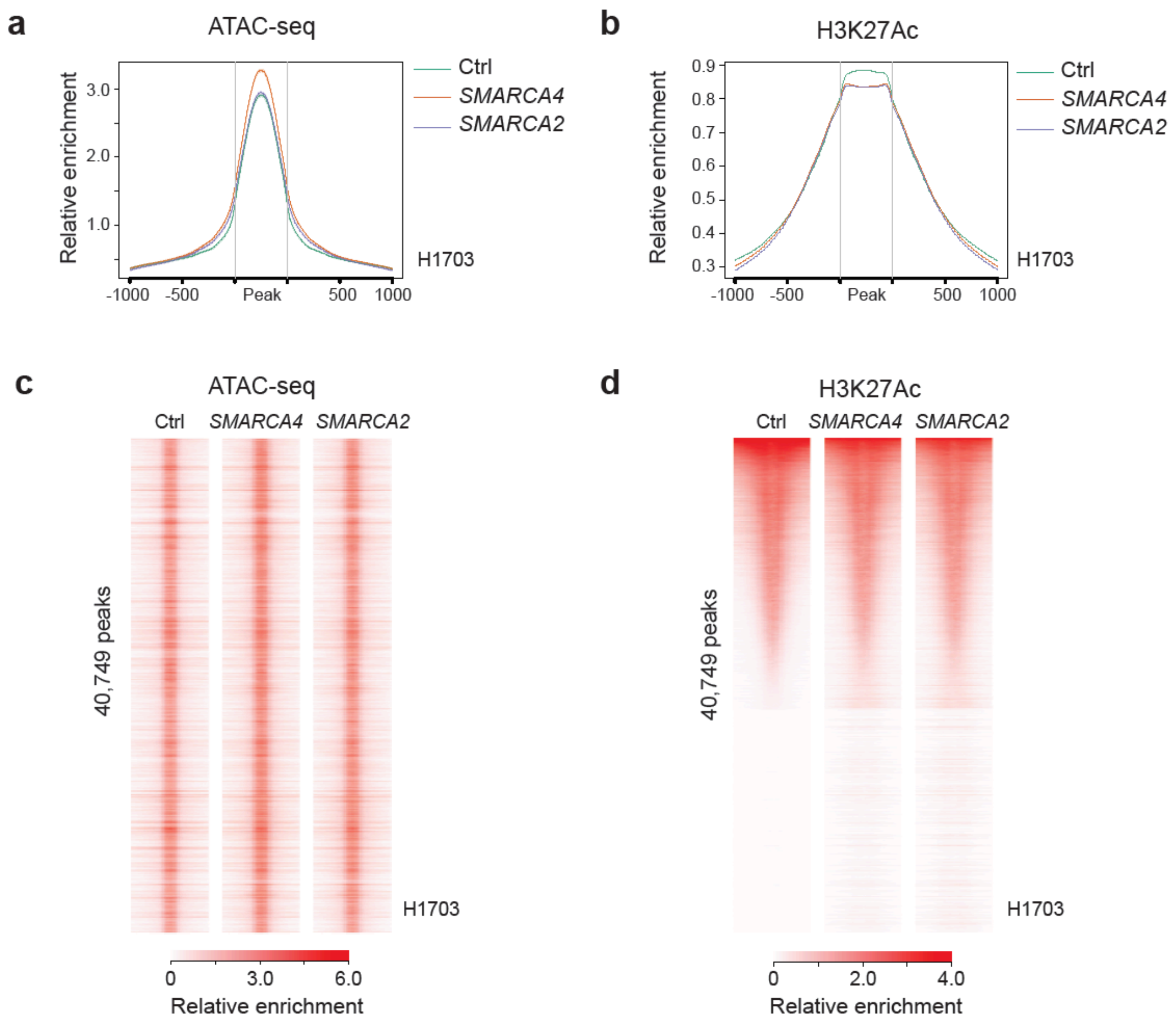

Supplementary Figure 10 | Similar levels of chromatin openness and H3K27 acetylation over conserved open regions.

a, c, Metaplot (**a**) and heatmap (**c**) of ATAC-seq read data from control, SMARCA4-restored and SMARCA2-restored cells over the 40,749 regions of conserved open chromatin between control and SMARCA2-restored cells as shown in the Venn diagram in Figure 4a.

b, d, Metaplot (**b**) and heatmap (**d**) of H3K27Ac ChIP data from control, SMARCA4-restored and SMARCA2-restored cells over over the 40,749 regions of conserved open chromatin between control and SMARCA2-restored cells.

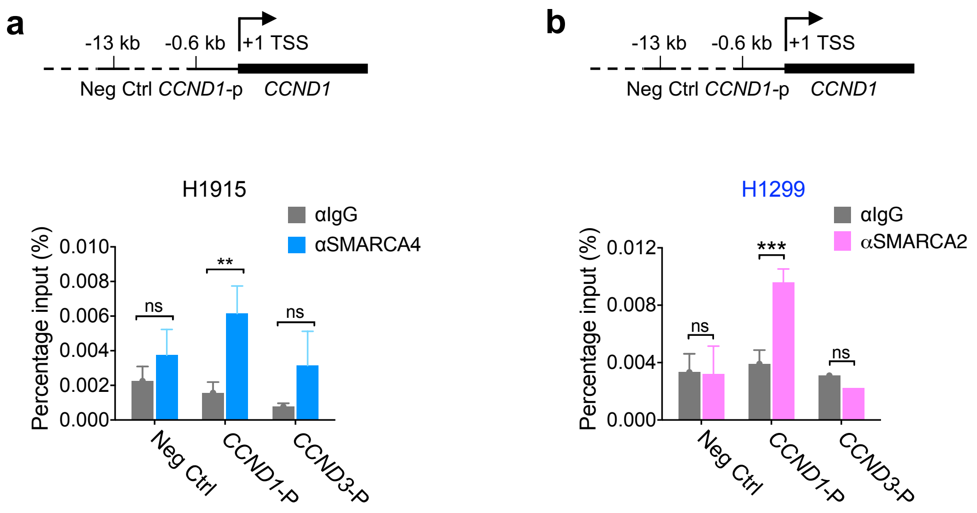

Supplementary Figure 11 | SMARCA4 and SMARCA2 bind to the promoter region of *CCND1* locus in NSCLC cells.

SMARCA4/2 occupancy in the promoter region of *CCND1* locus in NSCLC cells. Chromatin immunoprecipitation experiments were performed in SMARCA4/2-proficient H1915 cells using an antibody against SMARCA4 or IgG control (**a**). Chromatin immunoprecipitation experiments were performed in SMARCA4-deficient H1299 cells using an antibody against SMARCA2 or IgG control (**b**). TSS, transcription start site. Enrichment signals were quantified by qPCR with primers matching regions of negative control (Neg Ctrl, -13kb upstream of TSS), *CCND1* promoter (*CCND1*-P) and *CCND3* promoter (*CCND3*-P) as indicated. Error bars represent mean ± standard error of mean (s.e.m.); two-tailed *t*-test; ns, not significant; ** $p < 0.01$, *** $p < 0.001$.

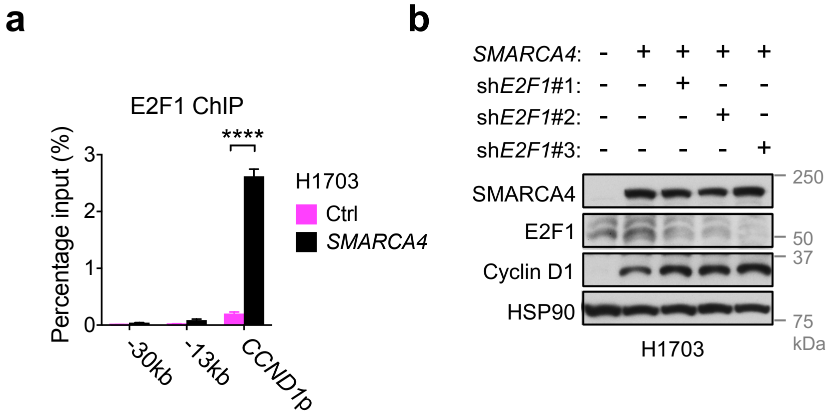

Supplementary Figure 12 | Role of E2F1 in the *CCND1* regulation by SMARCA4

a, Increased E2F1 occupancy at the *CCND1* promoter region in respond to SMARCA4 restoration in H1703 cells, suggesting increased chromatin accessibility at this locus induced by SMARCA4. Chromatin immunoprecipitation experiments were performed in H1703 cells before and after restoration of SMARCA4 using an antibody against E2F1. Enrichment signals were quantified by qPCR with primers matching regions of -30kb, -13kb upstream of TSS or *CCND1* promoter (*CCND1*-P) as indicated. Error bars represent mean ± standard error of mean (s.e.m.); two-tailed *t*-test; **** $p < 0.0001$.

b, Western blots show that E2F1 is not regulated by SMARCA4 and E2F1 knockdown results in slight upregulation of cyclin D1 expression in H1703 cells.

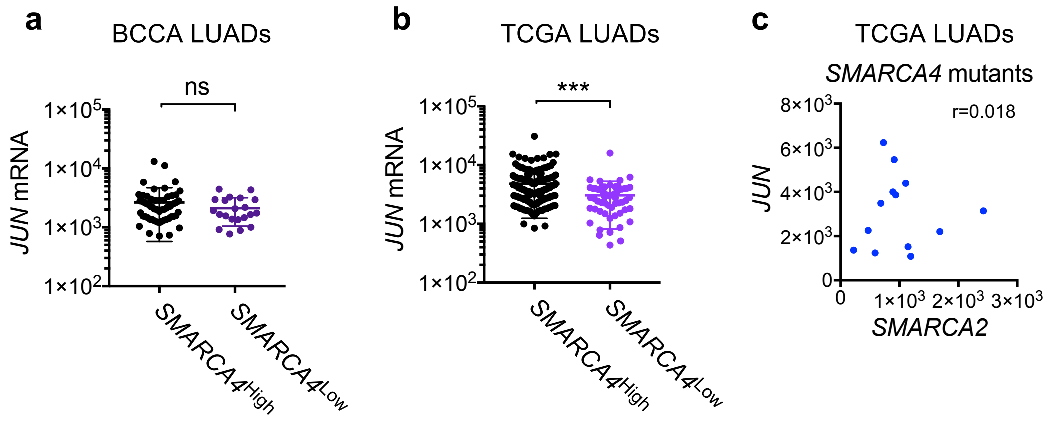

Supplementary Figure 13 | Correlation between *SMARCA4/2* and *JUN* mRNA expressions in lung cancer patient tumors

a, b, Correlation of *JUN* and *SMARCA4* mRNA expression in two cohorts of lung adenocarcinomas (LUADs) from BC Cancer Agency (BCCA; n = 83, **a**) and The Cancer Genome Atlas (TCGA; n = 230, **b**). SMARCA4^{Low}: the lower quartile of SMARCA4 mRNA expression in these tumors; SMARCA4^{High}: the rest. Error bars represent mean ± standard deviation (s.d.); two-tailed *t*-test; ns, not significant; *** p < 0.001.

c, Correlation of *CCND1* and *SMARCA2* mRNA expression in SMARCA4 mutated LUADs (n = 13) in the TCGA cohort. r, Pearson correlation coefficient.

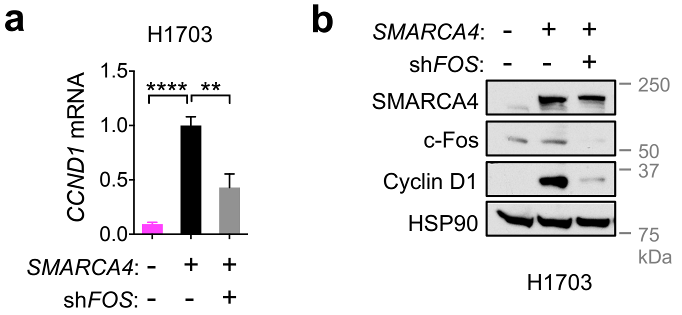

Supplementary Figure 14 | Cyclin D1 expression induced by SMARCA4 partially requires c-Fos

Knockdown of *FOS* partially abrogated SMARCA4-mediated induction of cyclin D1 mRNA (**a**) and protein (**b**) expression in H1703 cells.

Two-tailed *t*-test. Error bars represent mean ± s.d., ** p < 0.01, **** p < 0.0001.

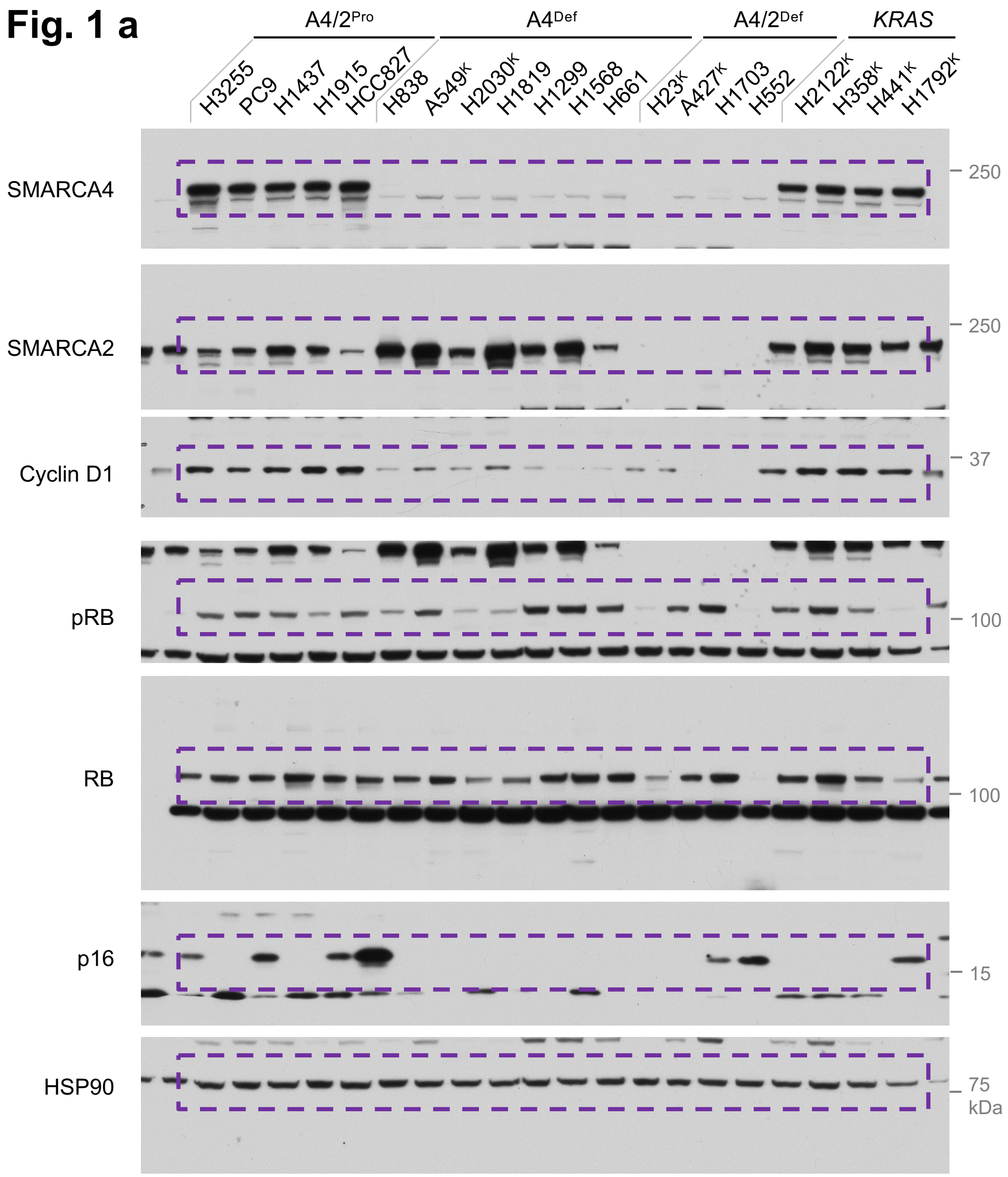

Supplementary Figure 15a | Uncropped scans for the key Western blots in Figure 1a.

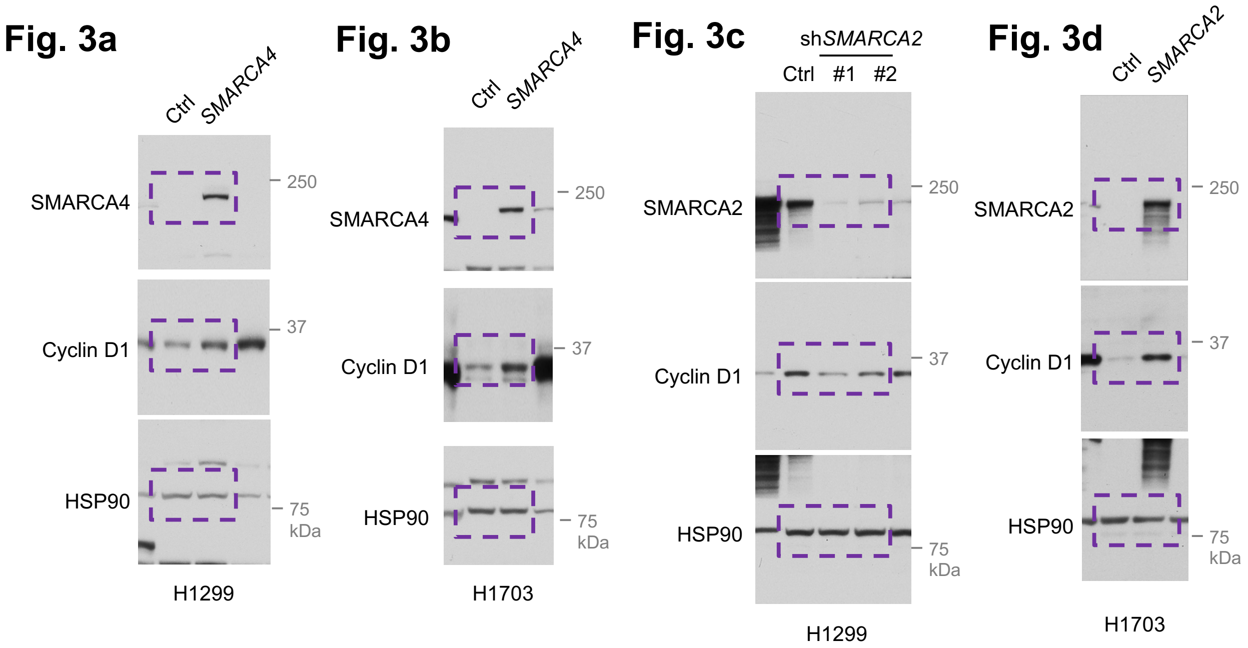

Supplementary Figure 15b | Uncropped scans for the key Western blots in Figure 3.

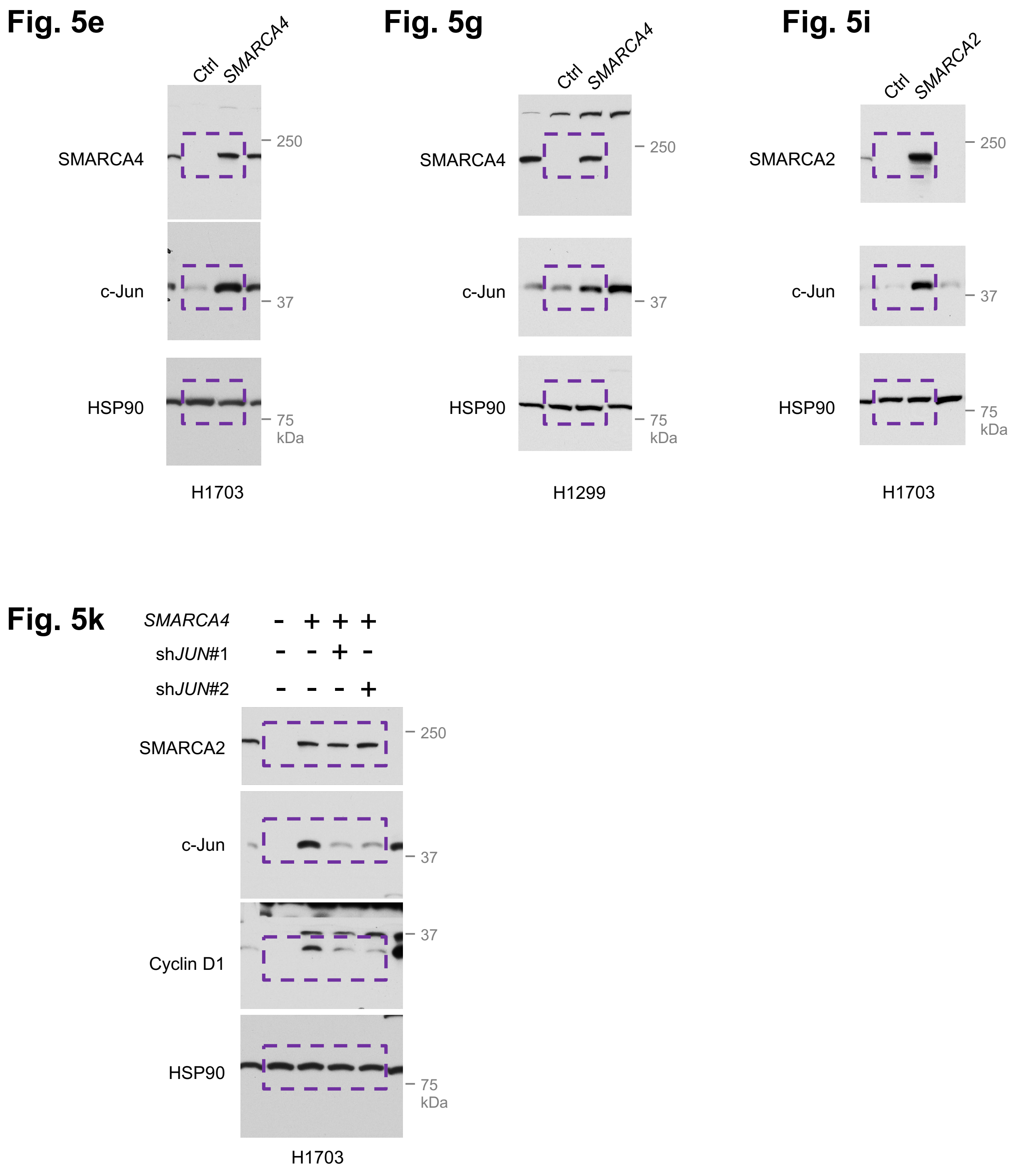

Supplementary Figure 15c | Uncropped scans for the key Western blots in Figure 5.
