# Supplementary material for: SMARCA4 loss is synthetic lethal with CDK4/6 inhibition in non-small cell lung cancer
Source: Nat Commun. 2019 Feb 4;10:557. doi: 10.1038/s41467-019-08380-1 (PMC6362083; doi:10.1038/s41467-019-08380-1)
Supplement: Supplementary file 2 — Description of Additional Supplementary Files [file 41467_2019_8380_MOESM2_ESM.docx]

**Description of Additional Supplementary Files**

File Name: Supplementary Data 1

Description: Mutation status of known oncogenic driver genes in the panel of NSCLC cell lines.

Mutation status of each cell line for listed genes were generated from Catalogue Of Somatic Mutations In Cancer (COSMIC). A4/2 Pro: SMARCA4/2 Proficient cell lines; A4 Def: SMARCA4 Deficient cell lines; A4/2 Def: SMARCA4/2 Deficient cell lines; KRAS: Cell lines with KRAS mutation; WT: wild type.

File Name: Supplementary Data 2

Description: Immunohistochemistry analysis results of SMARCA4, cyclin D1, RB and p16 in NSCLC patient tumors.

For SMARCA4 and RB1, unequivocally absent staining in the nuclei of viable tumor cells as opposed to strong staining in background stromal cells was considered as LOSS. P16 was analyzed according to the staining intensity on a scale of 0-3 (0 = negative, 1 = weak, 2 = moderate, 3 = strong). Cyclin D1 staining results were presented as H-scores.

File Name: Supplementary Data 3

Description: Descriptions of and mapping statistics for sequencing libraries generated.

For each ChIP-seq and ATAC-seq sample, the number of reads sequenced, mapped, and removed as duplicates is indicated. For ATAC-seq samples, reads that mapped to multiple locations in the genome were also removed. The final unique reads were used in all subsequent analysis.

File Name: Supplementary Data 4

Description: Locations of ATAC-seq peaks.

The chromosomal locations of ATAC-seq peaks in the control and SMARCA4-expressing H1703 cells are indicated. Regions specific to one set or contained within the intersect of the two sets (see Figure 4A) are also listed. Each peak-set is listed in a separate tab of the spreadsheet.
