# Supplementary material for: SMARCA4 loss is synthetic lethal with CDK4/6 inhibition in non-small cell lung cancer
Source: Nat Commun. 2019 Feb 4;10:557. doi: 10.1038/s41467-019-08380-1 (PMC6362083; doi:10.1038/s41467-019-08380-1)
Supplement: Supplementary file 8 — Reporting Summary [file 41467_2019_8380_MOESM8_ESM.pdf]

nature research

Corresponding author(s): Sidong Huang

Reporting Summary

Nature Research wishes to improve the reproducibility of the work that we publish. This form provides structure for consistency and transparency in reporting. For further information on Nature Research policies, see Authors & Referees and the Editorial Policy Checklist.

Statistical parameters

When statistical analyses are reported, confirm that the following items are present in the relevant location (e.g. figure legend, table legend, main text, or Methods section).

n/a	Confirmed	
☐	☒	The exact sample size (n) for each experimental group/condition, given as a discrete number and unit of measurement
☐	☒	An indication of whether measurements were taken from distinct samples or whether the same sample was measured repeatedly
☐	☒	The statistical test(s) used AND whether they are one- or two-sided<br>*Only common tests should be described solely by name; describe more complex techniques in the Methods section.*
☐	☒	A description of all covariates tested
☐	☒	A description of any assumptions or corrections, such as tests of normality and adjustment for multiple comparisons
☐	☒	A full description of the statistics including central tendency (e.g. means) or other basic estimates (e.g. regression coefficient) AND variation (e.g. standard deviation) or associated estimates of uncertainty (e.g. confidence intervals)
☐	☒	For null hypothesis testing, the test statistic (e.g. F, t, r) with confidence intervals, effect sizes, degrees of freedom and P value noted<br>*Give P values as exact values whenever suitable.*
☒	☐	For Bayesian analysis, information on the choice of priors and Markov chain Monte Carlo settings
☒	☐	For hierarchical and complex designs, identification of the appropriate level for tests and full reporting of outcomes
☐	☒	Estimates of effect sizes (e.g. Cohen's d, Pearson's r), indicating how they were calculated
☐	☒	Clearly defined error bars<br>*State explicitly what error bars represent (e.g. SD, SE, CI)*

Our web collection on statistics for biologists may be useful.

Software and code

Policy information about availability of computer code

Data collection	No software was used for data collection.
Data analysis	Methods, Patient tumor samples/mRNA expression analysis, Statistical analysis

For manuscripts utilizing custom algorithms or software that are central to the research but not yet described in published literature, software must be made available to editors/reviewers upon request. We strongly encourage code deposition in a community repository (e.g. GitHub). See the Nature Research guidelines for submitting code & software for further information.

Data

Policy information about availability of data

All manuscripts must include a data availability statement. This statement should provide the following information, where applicable:

- Accession codes, unique identifiers, or web links for publicly available datasets
- A list of figures that have associated raw data
- A description of any restrictions on data availability

All data are contained within this manuscript, and source data will be archived before publication or available through contacting the corresponding authors.

Field-specific reporting

Please select the best fit for your research. If you are not sure, read the appropriate sections before making your selection.

☒ Life sciences ☐ Behavioural & social sciences ☐ Ecological, evolutionary & environmental sciences

For a reference copy of the document with all sections, see nature.com/authors/policies/ReportingSummary-flat.pdf

Life sciences study design

All studies must disclose on these points even when the disclosure is negative.

Sample size	Described in Methods and Legends
Data exclusions	TMA cores with low tumour cellularity and artifacts were not included in the analysis. This was pre-established. See Methods.
Replication	All experiments were repeated for at least three times successfully.
Randomization	Mice with tumours of similar size were housed in groups of 3-5 and were randomly assigned to either vehicle control or treatment groups on Day 1 of treatment. Methods, Mouse xenografts, patient-derived xenografts and in vivo drug studies
Blinding	The person administering the drug or placebo was not blinded to the drug condition. However, the subsequent measurements and IHC analysis were blinded to the treatment information. Methods, Mouse xenografts and in vivo drug studies.

Reporting for specific materials, systems and methods

Materials & experimental systems

n/a	Involved in the study
☒	☐ Unique biological materials
☐	☒ Antibodies
☐	☒ Eukaryotic cell lines
☒	☐ Palaeontology
☐	☒ Animals and other organisms
☐	☒ Human research participants

Methods

n/a	Involved in the study
☒	☐ ChIP-seq
☒	☐ Flow cytometry
☒	☐ MRI-based neuroimaging

Antibodies

Antibodies used	Methods, Compounds and antibodies
Validation	Validated antibodies against HSP90 (H-114), Cyclin D1 (A12), CDK6 (C-21), CDK4 (DCS-35), p16 (C-20), p21 (H164), p27 (C19) and cyclin E (HE12) were from Santa Cruz Biotechnology; antibodies against Cyclin D2 (D52F9) and p-RB (S795) were from Cell Signaling; antibody against SMARCA4 were from Bethyl Laboratories (A300-813A). Antibody against Rb (554136) was from BD Pharmingen. Cyclin D3 (ab28283) antibody was from Abcam. Antibodies for ChIP: IgG (abcam ab37415), SMARCA4 (Bethyl A300-813A), SMARCA2 (Cell Signaling, D9E8B). Antibodies for mouse tumor IHC: Phosphor RB (Cell Signaling, 9308) and KI-67 (Abcam, 16667). Antibodies for patient tumor IHC: SMARCA4 (Abcam, clone EPNCIR111A; Santa Cruz Biotechnology, clone sc-17796), p16 (BD Pharmingen, clone G175-405) and RB1 (BD Pharmingen, G3-245), Cyclin D1 (Cell Marque - Sigma Aldrich, clone SP4).

Eukaryotic cell lines

Policy information about cell lines

Cell line source(s)	Methods, Cell culture and and Viral Transduction
Authentication	Cell lines were purchased from ATCC or authenticated with STR-GenePrinter.
Mycoplasma contamination	All cell line were tested and mycoplasma free. Methods, Cell culture and and Viral Transduction.

Commonly misidentified lines (See ICLAC register)	None.

Animals and other organisms

Policy information about studies involving animals; ARRIVE guidelines recommended for reporting animal research

Laboratory animals	Methods, Mouse xenografts and in vivo drug studies.
Wild animals	This study did not involve wild animals.
Field-collected samples	This study did not involve samples collected from the field.

Human research participants

Policy information about studies involving human research participants

Population characteristics	Methods, Patient tumor samples
Recruitment	Methods, Patient tumor samples
